# Supplementary material for: Crucial Role of FABP3 in αSyn-Induced Reduction of Septal GABAergic Neurons and Cognitive Decline in Mice
Source: Int J Mol Sci. 2021 Jan 1;22(1):400. doi: 10.3390/ijms22010400 (PMC7795765; doi:10.3390/ijms22010400)
Supplement: Supplementary file 1 [file ijms-22-00400-s001.pdf]

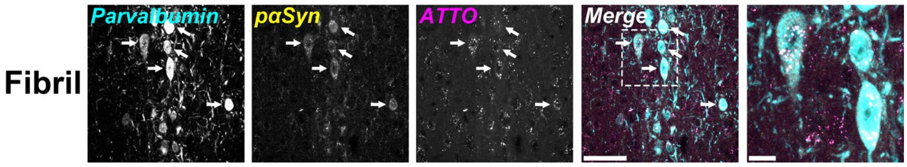

Figure S1. Accumulation of pαSyn in parvalbumin-positive neurons in the MS/nDB after intrastriatal injection of αSyn. Representative images of pαSyn (yellow) accumulation and exogenously applied αSyn (ATTO; magenta) in parvalbumin-positive cells (cyan) in the MS/nDB 30 days after the injection of αSyn fibrils. Scale bar: 50 (merge) and 10 (magnification) μm, respectively. Arrowheads depict cells positive for both pαSyn and parvalbumin.

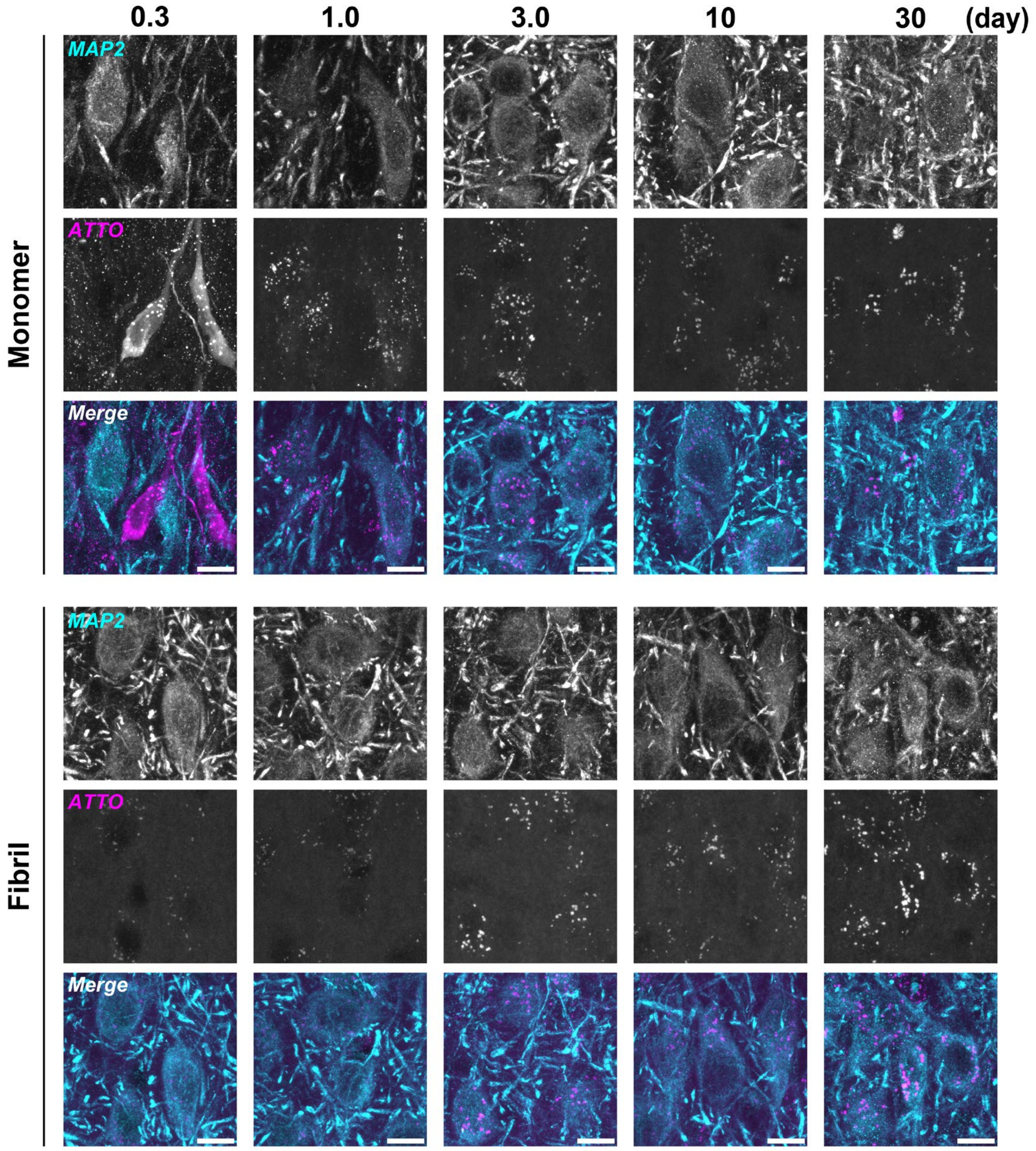

Figure S2. Temporal profile of the internalization of exogenous αSyn in neurons in the MS/nDB after intrastriatal injection of αSyn monomers and fibrils. Representative images of accumulation of exogenously applied *α*Syn (ATTO; magenta) in MAP2-positive neurons (cyan) in the MS/nDB at indicated days after the injection. Scale bar: 10 µm.

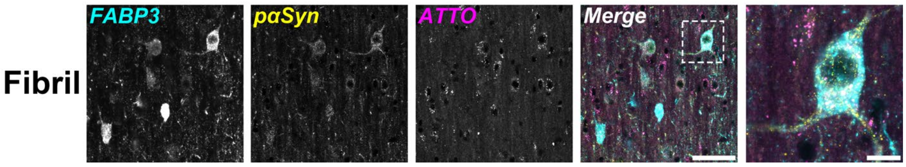

Figure S3. Accumulation of pαSyn in FABP3-positive neurons in the MS/nDB after intrastriatal injection of αSyn. Representative images of pαSyn (yellow) accumulation and exogenously applied αSyn (ATTO; magenta) in FABP3-positive cells (cyan) in the MS/nDB 30 days after the injection of αSyn fibrils. Scale bar: 50 (merge) and 10 (magnification) μm, respectively.

Table S1. Antibodies used in this study. IB, immunoblotting; IF, immunofluorescence.

Antibody	Supplier	RRID_AB	Dilution
αSyn (rodent-specific)	Cell Signaling Technology, Danvers, MA, USA	1904156	1:1000 (IF/IB)
β-actin	Sigma-Aldrich, St. Louis, MO, USA	476697	1:10000 (IB)
ChAT	Millipore, Burlington, MA, USA	2079751	1:1000 (IF)
FABP3	Hycult Biotech, Uden, Netherlands	533050	1:1000 (IF)
FABP3	Proteintech, Rosemont, IL, USA	2102309	1:3000 (IB)
GAD67	Millipore	2278725	1:1000 (IF)
GAD67	Abcam, Cambridge, UK	1310248	1:500 (IF)
MAP2	Abcam	2138153	1:500 (IF)
Parvalbumin	Swant, Marly, Swiss	10000343	1:2000 (IF)
pαSyn (S129)	Abcam	869973	1:2000 (IF)
Synaptophysin	Sigma-Aldrich	477523	1:500 (IF)
Alexa 405 anti-mouse	Abcam	2687445	1:600 (IF)
Alexa 405 anti-goat	Abcam	2636888	1:600 (IF)
Alexa 488 anti-mouse	Invitrogen, Waltham, MA, USA	2534069	1:600 (IF)
Alexa 488 anti-rabbit	Invitrogen	2535792	1:600 (IF)
Alexa 488 anti-goat	Invitrogen	2534102	1:600 (IF)
Alexa 488 anti-chicken	Jackson ImmunoResearch, West Grove, PA, USA	2340375	1:600 (IF)
Alexa 594 anti-rabbit	Invitrogen	141637	1:600 (IF)
Alexa 594 anti-goat	Invitrogen	2534105	1:600 (IF)
Alexa 594 anti-chicken	Jackson ImmunoResearch	2340377	1:600 (IF)
Biotin anti-mouse	Jackson ImmunoResearch	2307438	1:1000 (IF)
HRP anti-mouse	Southern Biotech, Birmingham, AL, USA	2619742	1:10000 (IB)
HRP anti-rabbit	Southern Biotech	2632593	1:10000 (IB)
